# P-619. Effectiveness of the 10-valent Pneumococcal Conjugate Vaccine (PCV10) on Hospital Admission due to Pneumonia among Nepalese Children

**DOI:** 10.1093/ofid/ofae631.817

**Published:** 2025-01-29

**Authors:** Yumiko Hayashi, Dhruba Shrestha, Ganendra Raya, Raj Shrestha, Konosuke Morimoto, Christopher Parry, Koya Ariyoshi, Bhim G Dhoubhadel

**Affiliations:** School of Tropical Medicine and Global Health, Nagasaki University, Nagasaki, Nagasaki, Japan; Siddhi Memorial Hospital, Bhaktapur, Bagmati, Nepal; Siddhi Memorial Hospital, Bhaktapur, Bagmati, Nepal; Siddhi Memorial Hospital, Bhaktapur, Bagmati, Nepal; Institute of Tropical Medicine, Nagasaki University, Nagasaki, Nagasaki, Japan; Liverpool School of Tropical Medicine, Liverpool, England, United Kingdom; Institute of Tropical Medicine, Nagasaki University, Nagasaki, Nagasaki, Japan; Institute of Tropical Medicine, Nagasaki University, Nagasaki, Nagasaki, Japan

## Abstract

**Background:**

*Streptococcus pneumoniae* is the most common bacteria that causes pneumonia in children. The 10-valent pneumococcal vaccine (PCV10) has been introduced in the national immunization program (2+1 schedule: 6 and 10 weeks, and 9 months) in Nepal since 2015. The impact of PCV10 on hospital admission due to pneumonia is not well known in Nepal.

**Methods:**

A cross-sectional study was carried out in Siddhi Memorial Hospital (SMH), Bhaktapur, Nepal, including hospital data from 2014 to 2022. SMH is the only pediatric general hospital in Bhaktapur district. The impact of PCV10 was assessed as a prevalence ratio analyzed with a log-binomial regression model. The prevalence ratio was calculated with the prevalence of hospital-admitted children due to chest x-ray confirmed pneumonia before and after the introduction of PCV10. The adjusted prevalence ratio (aPR) was measured in each year of the post-PCV10 period (2016 to 2022) by comparing the pre-PCV10 period (2014 and 2015) and adjusted by age, sex, and month of admission.

**Results:**

A total of 10,897 children were admitted to the hospital during the study period (2014-2022). The prevalence of pneumonia was 20.4% (429/2,105) in the pre-PCV10 period and 10.5% (923/8,792) in the post-PCV10 period. The overall aPR declined; it was 0.50 (95%CI: 0.42-0.59) in 2019, just before the COVID-19 pandemic, and 0.32 (95%CI: 0.26-0.40) in 2022. Among children of 2 months to 2 years of age, we observed a 51.7% reduction in hospital admission due to pneumonia (aPR: 0.48, 95%CI: 0.37-0.61) in 2019 and a 72.6% reduction (aPR: 0.27, 95%CI: 0.19-0.40) in 2022.

**Conclusion:**

We observed a significant decrease in hospital admissions due to pneumonia among children after the introduction of PCV10 in the national immunization program in Nepal. However, the surveillance of cases of pneumonia should be continued as the non-vaccine pneumococcal serotypes may emerge and hospital admissions due to pneumonia may go up in the future.

**Disclosures:**

**Konosuke Morimoto, MD, PhD**, Pfizer: Grant/Research Support **Koya Ariyoshi, MD, PhD**, Pfizer: Grant/Research Support

